# Markedly increased small-sized megakaryocytes and platelets count in the circulation with pseudo-hyperkalemia following splenectomy

**DOI:** 10.1186/s40001-022-00787-9

**Published:** 2022-08-23

**Authors:** Guiying Li, Bin Wang, Dongyan Li, Rong Zhu, Xueyan Chen

**Affiliations:** 1 Department of Clinical Laboratory, Inner Mongolia Xingan League People’s Hospital, Ulanhot, the Inner Mongolia Autonomous Region China; 2 Clinical Laboratory, Department of Clinical Laboratory, The People’s Hospital of Longhua Shenzhen, Shenzhen, 518109 China

## Abstract

Megakaryocytes are common in the bone marrow and appear less often in circulation. Most studies on circulatory megakaryocytes have implicated myelodysplastic syndromes and myeloproliferative disorders because of disruption of the bone marrow barrier and extramedullary hematopoiesis that is commonly seen in the spleen. As myeloproliferative disorders progress, particularly in the absence of the spleen, it is very likely that considerable numbers of megakaryocytes are present in the circulation. Myeloproliferation is associated with essential thrombocytosis or leukocytosis and is the leading cause of pseudo-hyperkalemia followed by reactive thrombocytosis due to splenectomy, rheumatoid arthritis, and renal cancer. The simultaneous measurement of plasma potassium is required when the platelet count exceeds 500 × 10^9^/L and the level of serum potassium is  > 5.4 mmol/L.

Dear Sir,

A 65-year-old male patient was hospitalized due to liver cirrhosis with ascites and complained of asthenia, dyspnea, and headache. He had a 5-year medical history of JAK2V617F mutant primary myelofibrosis. Bone marrow biopsy at initial diagnosis showed myelofibrosis with a severe increase in reticulin fibers (grade 4). The patient stopped taking thalidomide and was admitted to the hospital several times with recurrent abdominal distension and abdominal pain. Splenectomy was performed 1 year earlier because of concurrent hypersplenism and portal hypertension. It could not be excluded that the patient had progressed from primary myelofibrosis to leukemia combined with the poor general condition and abnormal clinical tests at admission. The patient died 5 days after hospitalization due to the worsening of their condition.

Biochemical examinations at the time of admission showed obvious abnormalities in some indices including albumin (32.8 g/L), lactate dehydrogenase (1521 U/L), blood urea nitrogen 16.02 (mmol/L), creatinine (115 µmol/L), uric acid (685. 9 µmol/L), serum potassium (6.19 mmol/L), triglycerides (2.67 mmol/L) and C-reactive protein (37.7 mg/L). Blood glucose levels were not significantly increased. The complete blood count on the XN-9000 analyzer showed a white cell count (WBC) of 14.51 × 10^9^/L (neutrophils 0%, lymphocytes 38%, blasts 4%, eosinophils 7%, basophils 0% and abnormal cells 51%), hemoglobin (Hb) 39 g/L, a platelet count from platelet electrical impedance channel 218 × 10^9^/L and nucleated red blood cells at 3/100 white cells. The abnormal cells were negative for myeloperoxidase.

Considering the abnormal blood results and the WBC scattergrams that merited re-examination, microscopic examinations were performed. Numerous small-sized megakaryocytes and prominent bare megakaryocytes were observed in the peripheral blood smears (Fig. 1) which accounted for 60% of the total nucleated cell count (TNCC). Superficially, these cells can be easily confused with atypical lymphoid cells due to the round nuclei, condensed chromatin, and rough or irregular cytoplasm. However, the cloud-like cytoplasm distinguishes small megakaryocytes from lymphocytes. The positive CD41 staining in the lymphocyte-like cells confirmed megakaryocytes. The true value of leukocytes was 9.6 × 10^9^/L. Also, there was a proportionately higher number of large and giant-sized platelets that lead to a spurious reduction in the platelet count with an actual count of 1676 × 10^9^/L after correction on the PLT fluorescence channel.

**Fig. 1:**
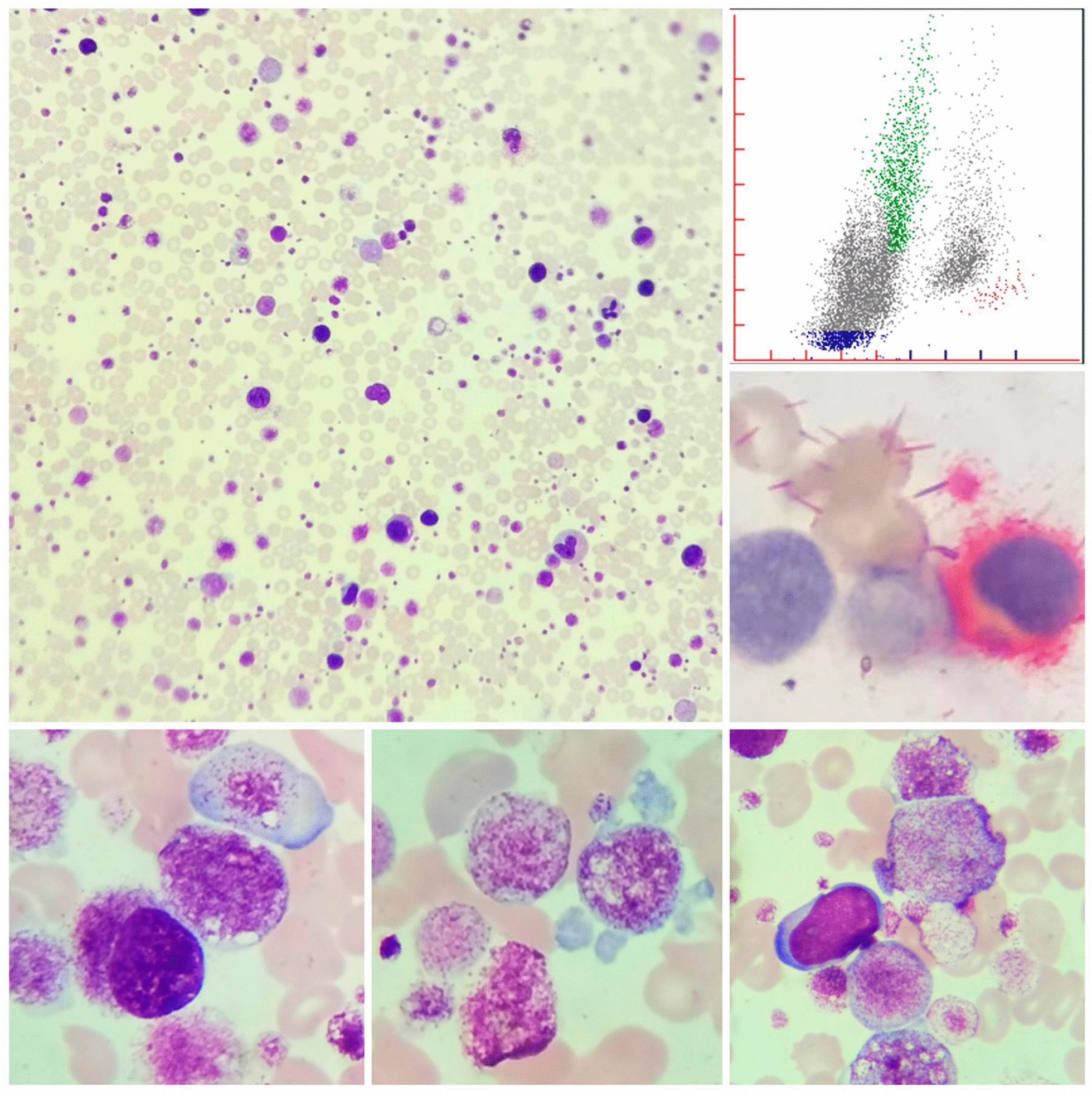
Peripheral blood smear (Wright–Giemsa stain, 40 × magnification) showing the presence of numerous small-sized megakaryocytes and giant platelets (top left); peripheral blood smear (Wright–Giemsa stain, 1000 × magnification) showing the presence of numerous small-sized megakaryocytes and giant platelets (bottom left, middle); peripheral blood smear (Wright–Giemsa stain, 1000 × magnification) showing myeloid blast (bottom right); the lymphocyte-like cells were positive for CD41 staining (middle right); abnormal WDF scatter plots of the patient. Gray scatter plots indicating the presence of abnormal cells (top right)

The patient did not have clinical manifestation or electrocardiographic signs of adverse cardiac events despite markedly increased serum potassium. No definitive reasons for the apparent and persistent increase in serum potassium were found. After reviewing the literature and consulting with a specialist, pseudo-hyperkalemia was not excluded. Finally, the concentration of plasma potassium was normal at 3.3 mmol/L.

Megakaryocytes are common in the bone marrow and appear less often in circulation. Most studies on circulatory megakaryocytes have implicated myelodysplastic syndromes and myeloproliferative disorders because of disruption of the bone marrow barrier and extramedullary hematopoiesis that is commonly seen in the spleen [1]. As myeloproliferative disorders progress, particularly in the absence of the spleen, it is very likely that considerable numbers of megakaryocytes are present in the circulation and can be counted using an automated multichannel hematology analyzer as the cells are similar in size to leukocytes [2].

The initial diagnosis in most patients with PMF occurs with marked fibrosis during which bone marrow biopsy shows marked proliferation of reticular or collagen fibers (fibrosis grade 2 or 3) [3]. Abnormal morphological findings such as large, degranulated platelets and teardrop-shaped red blood cells are often present in the peripheral blood in myelofibrosis sometimes with naked and small-sized megakaryocytes [3]. These megakaryocytes morphologically resemble lymphocytes and lack obvious morphological features that are easily overlooked by inexperienced pathologists. Circulating megakaryocytes are also readily found in RAEB cases, subtypes of myelodysplastic syndrome, and can migrate from the bone marrow into circulation to indicate more aggressive types of myelodysplastic syndrome [4]. The routine assessment of micro-megakaryocytes in peripheral blood smears can therefore be considered part of the clinical workup.

Based on a large sample size, Ong YL et al. concluded that acute and chronic myeloproliferative is associated with essential thrombocytosis or leukocytosis and is the leading cause of pseudo-hyperkalemia followed by reactive thrombocytosis due to splenectomy, rheumatoid arthritis, and renal cancer [5]. The case mentioned above involved a patient with primary myelofibrosis who had undergone a splenectomy illustrating that both primary and secondary hematological disease can contribute to this false situation. Splenectomy prevents the destruction of platelets resulting from hypersplenism and at the same time, thrombocytosis can be observed along with a slight increase in the production of platelets in the bone marrow [6].

Normally, potassium is released from platelets during the coagulation process in vitro resulting in a higher concentration of potassium in the serum compared to the plasma (0.36 ± 0.18 mmol/l) [7]. The definition of pseudo-hyperkalemia means that the serum potassium levels  > 0.4 mmol/L without clinical signs of electrolyte imbalance and ECG changes [5]. Serum potassium was obtained from biochemical tubes containing the pro-coagulants, whereas the plasma sample was acquired from tubes with anticoagulants such as sodium heparin. Sodium heparin stabilizes and inhibits the aggregation of platelets to avoid pseudo-hyperkalemia caused by the release of potassium. *Thurlow *et al. suggested that the simultaneous measurement of plasma potassium is required to ensure the correctness of results when the platelet count exceeds 500 × 10^9^/L and the level of serum potassium is  > 5.4 mmol/L [8]. This case highlights the need to recognize the link between unusual morphological features and biochemical abnormalities by the involved professionals.

## Data Availability

The data set supporting the results of this article is included within the article.
